# Neuromodulation therapies for diabetic neuropathy: mechanisms, efficacy, and impacts on nerve health: a narrative review

**DOI:** 10.3389/fneur.2026.1892683

**Published:** 2026-07-27

**Authors:** Ethan M. Larson, Brian D. Dalm, Kristy L. Townsend

**Affiliations:** Department of Neurosurgery, The Ohio State University, College of Medicine and Wexner Medical Center, Columbus, OH, United States

**Keywords:** diabetic peripheral neuropathy, dorsal root ganglion stimulation, neuromodulation, neuroregeneration, spinal cord stimulation

## Abstract

Diabetic peripheral neuropathy (DPN) is a highly prevalent complication characterized by progressive nerve degeneration and often refractory neuropathic pain. Since current pharmacologic interventions are largely palliative, can carry significant adverse effects, and fail to halt disease progression, disease-modifying treatments are urgently needed. This narrative review examines the potential of neuromodulation therapies, specifically spinal cord stimulation (SCS), dorsal root ganglion (DRG) stimulation, and peripheral nerve stimulation (PNS), as alternatives for both symptom management and physiological neuroregeneration. To assess the data behind these interventions, a literature search of PubMed and Google Scholar from inception through June 2026 was conducted, encompassing randomized controlled trials, observational cohorts, and mechanistic evaluations of adult patients with painful DPN. The synthesized clinical evidence demonstrates that more invasive modalities, particularly SCS and DRG stimulation, consistently achieve greater than 50% sustained pain reduction in refractory populations while significantly decreasing opioid reliance. Beyond providing robust analgesia, data indicate implantable neuromodulation actively engages key pathophysiological mechanisms to promote structural nerve repair, including downregulating neuroinflammation, modulating maladaptive glial reactivity, and restoring microvascular perfusion. This regenerative potential is supported by improvements in nerve conduction velocities (NCVs) and increased intraepidermal nerve fiber density following therapy. Mechanistically, DRG stimulation attenuates satellite glial cell activation, SCS facilitates nitric oxide-mediated vasodilation, and PNS stimulates Schwann cell activity to encourage axonal repair. While more research is needed, ultimately, neuromodulation represents a paradigm shift in the management of DPN, evolving from a palliative option into a promising biologically grounded, disease-modifying therapy. To fully realize this potential, future clinical trials must systematically incorporate objective structural biomarkers, such as functional neuroimaging and corneal confocal microscopy, to validate long-term neuroregeneration and optimize early intervention.

## 1 Introduction to DPN

Diabetic peripheral neuropathy (DPN) is one of the most common and debilitating complications of diabetes mellitus, affecting up to 70% of individuals with long-standing disease, depending on the population (1). DPN encompasses a spectrum of peripheral nerve disorders, with distal symmetric polyneuropathy being the most prevalent manifestation. Due to the polyneuropathy that underlies most cases of DPN, symptoms are complex and range from numbness, burning, tingling, pain, and motor loss. Clinically, DPN leads to progressive sensory loss, chronic pain, autonomic dysfunction, and motor impairments; outcomes that not only reduce quality of life, but also significantly increase the risk of foot ulcers, infections, and amputations (2).

The pathogenesis of DPN is multifactorial and complex, without a clear mechanistic etiology. Glucotoxicity, lipotoxicity, and inflammation are common hypotheses of disease onset and progression. Current research indicates DPN could arise from a combination of hyperglycemia-induced oxidative stress, mitochondrial dysfunction, impaired axonal transport, and microvascular ischemia. These pathological processes are hypothesized to converge in ways that lead to both axonal degeneration and/or demyelination, potentially compounded by diminished neurotrophic support and chronic metabolic dysregulation in neurons and associated glial cells (3). However, the underlying mechanisms remain incompletely understood, and multiple pathogenic pathways, such as metabolic, inflammatory, and lipotoxic processes, are actively being investigated. Despite a growing understanding of these interrelated mechanisms, effective disease-modifying therapies remain elusive, leaving a significant therapeutic gap.

DPN is correlated with chronic hyperglycemia, but it remains unclear if the hyperglycemia or accompanying inflammation and dyslipidemia are responsible for initiating a pathogenic cascade characterized by oxidative stress, mitochondrial dysfunction, and neurodegeneration (4, 5). These insults, compounded by metabolic dysregulation and a decline in neurotrophic factors, also lead to impaired Schwann cell metabolism and disrupted axon–glia interactions (6). Consequently, peripheral nerves can undergo both axonal degeneration and segmental demyelination. Understanding this structural failure is critical, as emerging neuromodulation therapies aim not only to mask symptoms but to facilitate neuroregeneration in the alleviation of symptoms.

Clinically, DPN manifests differently depending on the fiber type involved. Small-fiber injury often presents early, characterized by exaggerated sensory symptoms such as burning dysesthesias and pain. In contrast, large-fiber involvement typically results in negative symptoms, including numbness, loss of proprioception, and eventual motor deficits (7). Distinguishing between these phenotypes is essential for selecting appropriate stimulation targets.

Diagnostic assessments must align with these anatomical distinctions. Standard Nerve Conduction Studies (NCS) are the cornerstone for detecting large-fiber pathology, revealing reduced conduction velocities consistent with demyelination (8). However, NCS cannot detect small-fiber neuropathy (SFPN). Therefore, while clinical screening tools exist, objective quantification of small-fiber loss requires intraepidermal nerve fiber density (IENFD) assessment via skin biopsy, but this histological quantification often misses functional deficits and SFPN functional clinical diagnostics do not exist beyond limited assessments of sweat gland function (ex: QSART), or skin sensory testing (9). Therapeutic efficacy, particularly regarding small-fiber regeneration, needs to be measured using the appropriate modality.

Current pharmacologic options, including gabapentin, pregabalin, duloxetine, tapentadol, and topical capsaicin primarily aim to relieve symptoms. However, their efficacy is variable, and adverse effects such as dizziness, sedation, and gastrointestinal distress often limit long-term use and patient adherence (10–12). Furthermore, recent epidemiological data indicate that the concomitant prescription of gabapentinoids with opioids significantly increases the risk of opioid use disorder and fatal opioid-related overdoses, compounding the dangers of both drug classes (13). In some cases, opioids are prescribed for more severe pain, yet their long-term use in DPN remains controversial due to limited evidence of sustained benefit and the well-documented risks of tolerance, dependence, misuse, and opioid-induced hyperalgesia. The American Pain Society recommends that opioids be reserved only for refractory cases of neuropathic pain, emphasizing the need for multimodal, opioid-sparing strategies whenever possible (14). These limitations in pharmacologic therapies underscore the urgent need for alternative interventions that go beyond palliation to address the underlying nerve pathology. Taken together, pharmacological approaches do not carry the safety and efficacy profiles desired for treating DPN currently.

Neuromodulation therapies present a potential paradigm shift. Modalities like SCS, PNS (including transcutaneous), and DRG stimulation deliver targeted electrical impulses to modulate neural activity at key anatomical nodes. Importantly, accumulating evidence suggests that these approaches not only reduce pain but also influence glial activation, neuroinflammatory signaling, and potentially enhance structural nerve regeneration—indicating both symptomatic and disease-modifying benefits that warrant deeper investigation (15, 16).

Current literature, including recent meta-analyses and large retrospective cohorts, has rigorously established the analgesic efficacy and safety of neuromodulation in DPN (17–20). However, a synthesis of the regenerative mechanisms remained lacking. This review addresses that gap by focusing exclusively on existing molecular and cellular evidence for neuroregeneration, shifting the focus from palliation to potential reversal of nerve damage (21). We synthesize recent advances across clinical and translational studies, with a focus on how neuromodulatory strategies might reframe the treatment landscape by promoting both functional relief and neurobiological recovery.

## 2 Methods

A narrative literature review was conducted to synthesize current evidence on neuromodulation therapies for DPN, focusing on both established analgesic efficacy and underlying cellular mechanisms. We searched PubMed and Google Scholar for English-language studies published from inception through June 2026. The search utilized the PICO framework, focusing on adult patients with Painful DPN (P) receiving invasive or non-invasive peripheral or spinal neuromodulation (I), compared to conventional medical management, sham, or baseline (C), to evaluate analgesic efficacy and physiological nerve regeneration (O). Search terms included combinations of “diabetic neuropathy,” “spinal cord stimulation,” “dorsal root ganglion stimulation,” and “peripheral nerve stimulation.” We specifically excluded intracranial neurostimulation modalities (e.g., rTMS, tDCS) to prioritize interventions targeting the peripheral and spinal axis, where direct modulation of dorsal horn circuitry and peripheral nerve fibers may offer unique regenerative potential. To maintain a rigorous evaluation of physiological neuroregeneration and sustained analgesia, this review focuses primarily on modalities that directly target the affected somatosensory neuroaxis (SCS, DRG, PNS) alongside standard transcutaneous interventions (TENS). While other emerging neuromodulatory approaches, such as vagus nerve stimulation (VNS), scrambler therapy, and therapeutic ultrasound, expand the therapeutic landscape, they remain largely investigational for DPN or involve systemic rather than localized anatomical targeting, and are therefore discussed separately in Section 4.3.

## 3 Results of review

### 3.1 Clinical analgesic efficacy

Neuromodulation refers to techniques that use electrical stimulation to modulate neural circuit activity, and growing evidence suggests it may potentially reverse pathological changes in neurological diseases including DPN. Among the most promising strategies are SCS, DRG stimulation, and PNS/TENS, each targeting distinct anatomical and physiological components of the nervous system. The synthesis included a diverse range of randomized controlled trials (RCTs), observational cohorts, and pilot mechanistic studies.

### 3.2 Spinal cord stimulation (SCS)

SCS delivers electrical impulses to the dorsal columns via epidural leads, primarily from T8–L2, thereby activating large-diameter Aβ fibers that inhibit nociceptive C-fiber transmission in the dorsal horn. Rooted in the gate control theory of pain, this electrical interference serves as the primary mechanism for analgesia by functionally blocking ascending nociceptive C-fiber signals before they can be perceived by the brain (22). High-frequency (10 kHz) and burst-mode stimulation, designed to be paresthesia-free, improved patient comfort without sacrificing efficacy (23–27). RCTs demonstrated that SCS can achieve ≥50% pain reduction in up to 80% of patients, with benefits extending over 24 months, including enhanced mobility, improved sleep, and reduced analgesic reliance (28–35). Like all implantable neuromodulation systems, SCS is associated with procedural and device-related risks, including lead migration, infection (typically <10%), hardware malfunction, and, rarely, neurological complications. Beyond analgesia, SCS promoted microvascular perfusion and improved NCV, supported by increased nitric oxide synthase expression in dorsal horn neurons (16, 36). Real-time optimization using closed-loop systems, which modulate electrical stimulation output based on evoked compound action potentials from the dorsal horns of the spinal cord, further enhanced outcomes while maintaining safety. While these findings are encouraging, it is important to recognize that many supporting studies are limited by small sample sizes, lack of sham-controlled designs, and variable outcome reporting, which complicate the interpretation of efficacy and generalizability. Nevertheless, this underscores not only the potential of neuromodulation but also the critical need for more rigorous clinical trials to validate and refine its role in DPN management.

**Figure 1:**
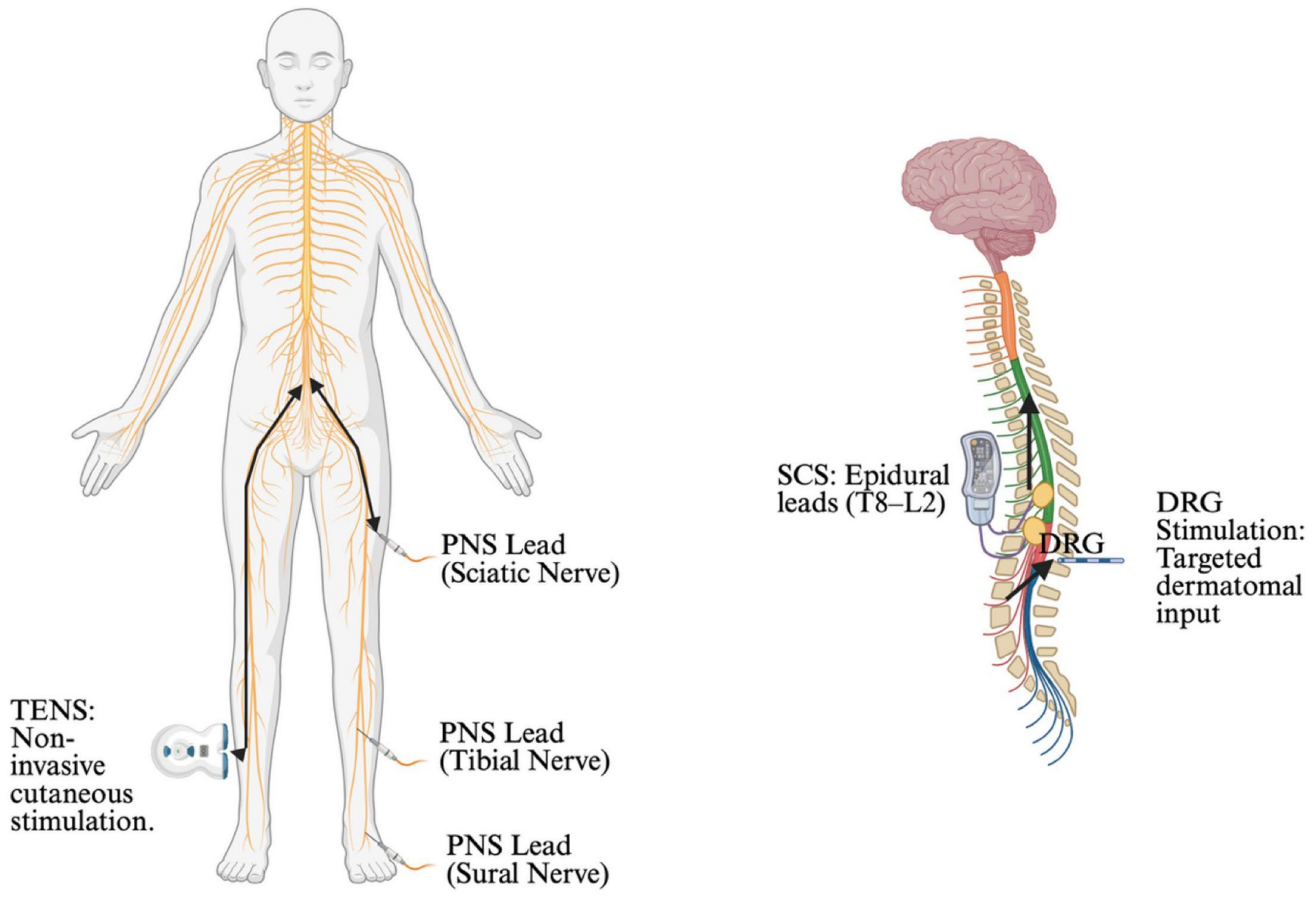
Anatomical targets of neuromodulation therapies in diabetic neuropathy. Left: Peripheral nerve targets of TENS, PNS, and their anatomical lead placements. Common PNS targets include the sciatic, tibial, and sural nerves. Right: Central targets of SCS and DRG stimulation. SCS leads are placed epidurally from T8–L2, while DRG electrodes interface with sensory ganglia for dermatomal specificity. These modalities engage both peripheral and central mechanisms of pain and nerve repair. Created with BioRender.com.

The options for neuromodulation therapies are illustrated in Figure 1, which depicts electrode placement for each modality. SCS leads are positioned over the dorsal columns, while DRG electrodes are placed adjacent to L2–L5 dorsal root ganglia. The figure also illustrates the anatomical zones targeted by PNS and TENS. This diagram helps contextualize how lead placement translates into dermatomal and nerve-specific effects, critical for individualized treatment. Consequently, a thorough clinical assessment, integrating pain topography, sensory phenotyping, and diagnostic NCVs, is essential to guide the decision-making process for selecting the most appropriate modality and its precise anatomical placement.

### 3.3 Dorsal root ganglion (DRG) stimulation

DRG stimulation targets the sensory neuron cell bodies at L2–L5, providing precise dermatomal pain relief without the paresthesia overlap often seen in SCS (15, 37). Unlike SCS, which provides more generalized regional coverage, DRG stimulation is specifically designed for highly localized, anatomically discrete pain, making it ideal for focal neuropathic symptoms that do not respond well to broader field stimulation. This means that the stimulation can be confined to the exact spinal level corresponding to the patient’s painful area, avoiding unintended sensations (paresthesias) in non-painful regions, a common issue with broader SCS coverage whereby stimulation can spill over into adjacent dermatomes and reduce targeting accuracy. The lower current requirements of DRG and more focused stimulation make it especially useful for patients with focal pain with DPN. Mechanistic studies revealed that DRG stimulation attenuated glial activation and excitatory signaling, while also supporting proprioceptive function, gait stability, and postural control (38–41). Notably, Figure 1 visually distinguishes DRG’s lateralized placement and its unique dermatomal reach, emphasizing its clinical precision compared to broader SCS coverage.

### 3.4 Peripheral nerve stimulation (PNS)/transcutaneous electrical nerve stimulation (TENS)

TENS is an accessible, non-invasive method that uses surface electrodes to stimulate Aβ fibers, thereby engaging segmental inhibition and descending analgesia. While TENS is safe and cost-effective, its clinical utility is often restricted by the short duration of its analgesic effect and the frequent development of neural habituation (42–44). Because TENS relies on temporary ‘gate control’ mechanisms and does not actively drive the neurotrophic or glial-modulating pathways seen in SCS or DRG stimulation, it provides symptomatic palliation without the sustained disease-modifying benefits observed in invasive therapies.

PNS directly targets peripheral nerves such as the tibial, sciatic, and sural nerves via percutaneous or implanted leads. Clinical trials report 60–80% pain relief and improved NCVs within 6–12 months (45, 46). PNS was also shown to stimulate Schwann cell activity, axonal repair, and promote regional wound healing, particularly when applied to the tibial nerve (44, 47). Because DPN inherently damages the large myelinated A-beta fibers typically targeted by PNS, future research must clarify whether higher stimulation amplitudes or altered programming strategies are required to achieve clinical analgesia and nerve regeneration in this specific population. To complement these findings, Table 1 summarizes each modality by mechanism, anatomical site, duration of effect, and regenerative potential. It contrasts the broad influence of SCS with the anatomically specific effects of DRG and PNS, while highlighting the transient nature of TENS. Table 2 extends this by aligning specific neuromodulation techniques with their dermatomal and peripheral nerve targets, offering a practical guide for lead positioning and treatment matching based on symptom distribution.

**Table 1:** Comparison of neuromodulation modalities in diabetic neuropathy.

Modality	Pain relief efficacy	Durability of clinical relief	Paresthesia-free options	Best use case	Mechanism (evidence)
Spinal cord stimulation (SCS)	High (60–80%)	>24 months	Yes (10 kHz or burst)	Diffuse bilateral lower limb neuropathy	Gate control + glial/cortical modulation
Dorsal root ganglion (DRG)	High (70–85%)	>24 months	Yes	Focal, unilateral or asymmetric pain distributions	DRG neural-glial modulation + gene expression
Peripheral nerve stimulation (PNS)	Moderate (50–70%)	6–12 months	Yes (implantable or ultrasound-guided)	Focal pain with preserved nerve conduction	Schwann regeneration + NCV improvement
Transcutaneous electrical nerve stimulation (TENS)	Low–Moderate (30–50%)	Weeks–Months	Yes	Early-stage DPN or adjunct for mild symptoms	Segmental inhibition + endogenous opioid/5-HT pathways

This table compares key neuromodulation modalities (SCS, DRG, PNS, TENS) across mechanism of action, anatomical targets, and clinical profile. “Durability of Clinical Relief” refers to the sustained maintenance of pain reduction over time (e.g., potential for tolerance or “washout” upon cessation). Data are derived from a synthesis of clinical trials and long-term observational studies.

**Table 2:** Nerve targets and dermatomal coverage by neuromodulation technique.

Modality	Primary target nerves	Access approach	Anatomical scope
SCS	Dorsal columns (T8–L2), modulating lumbar/sacral sensory	Epidural lead placement at thoracic spine	Broad lumbosacral region via dorsal column relay
DRG	Dorsal root ganglia (L2–S1) matching dermatomal zones	Transforaminal lead placement adjacent to DRG	Dermatomal-specific limb modulation
PNS	Peripheral nerves: tibial, sciatic, sural, peroneal	Percutaneous or surgical lead near the nerve trunk	Localized stimulation (foot, calf, thigh)
TENS	Superficial sensory nerves: saphenous, sural, common peroneal	Surface electrodes over dermatomal zones	Subcutaneous field targeting superficial sensory nerves

This table maps neuromodulation techniques to their anatomical targets, highlighting corresponding dermatomes and nerve territories. It emphasizes the precision of DRG stimulation and the broader targeting of SCS and PNS. This reference aids in lead placement planning and patient selection based on symptom distribution.

**Figure 2:**
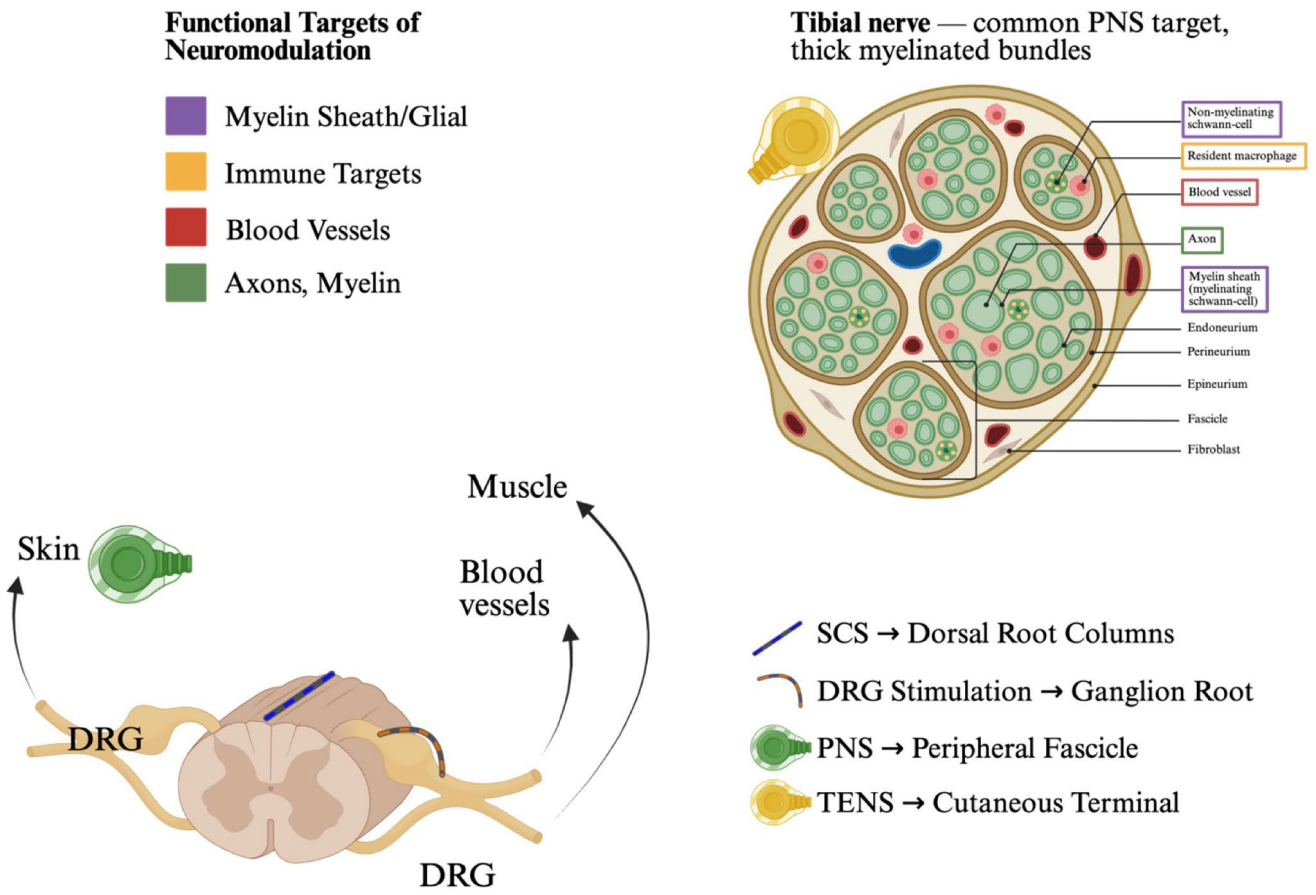
Peripheral targets and cellular architecture of neuromodulation in diabetic neuropathy. Left: Afferent sensory neuron originating from the dorsal root ganglion (DRG), projecting to peripheral targets including skin, muscle, and vascular structures. Right: Cross-sections of the tibial nerve (top; large-caliber, commonly targeted by PNS) and sural nerve (bottom; small-caliber, frequently sampled in skin biopsy). Labeled structures include axons, Schwann cells, resident macrophages, and vascular elements—key anatomical structures impacted by injury or DPN and implicated in potential repair mechanisms in diabetic neuropathy. Color-coded annotations highlight potential cellular targets of neuromodulation therapies (neuronal, glial, immune, and vascular). Created with BioRender.com.

The visual and anatomical insights from Figure 1 are expanded in Figure 2, which shifts to a microscopic perspective. Here, a cross-sectional schematic of peripheral nerves (tibial, sciatic, and sural) highlights how data from neuromodulation studies indicate axonal regrowth, myelin repair, and Schwann cell activity. This visual contextualization emphasizes that neuromodulation impacts may extend beyond symptom control, it actively engages biological repair mechanisms. Neuromodulation is not a monolithic intervention, but rather a spectrum of approaches with distinct anatomical targets, mechanisms of action, and therapeutic timelines. Additionally, neuromodulation is poorly understood mechanistically, and most proposed mechanisms of symptom relief are not sufficiently supported by rigorous human research, leaving open the likelihood that additional mechanisms of action are at play – including direct impacts on neuroprotection, neuroplasticity, and neuroregeneration that may halt and reverse the nerve damage that led to DPN.

**Figure 3:**
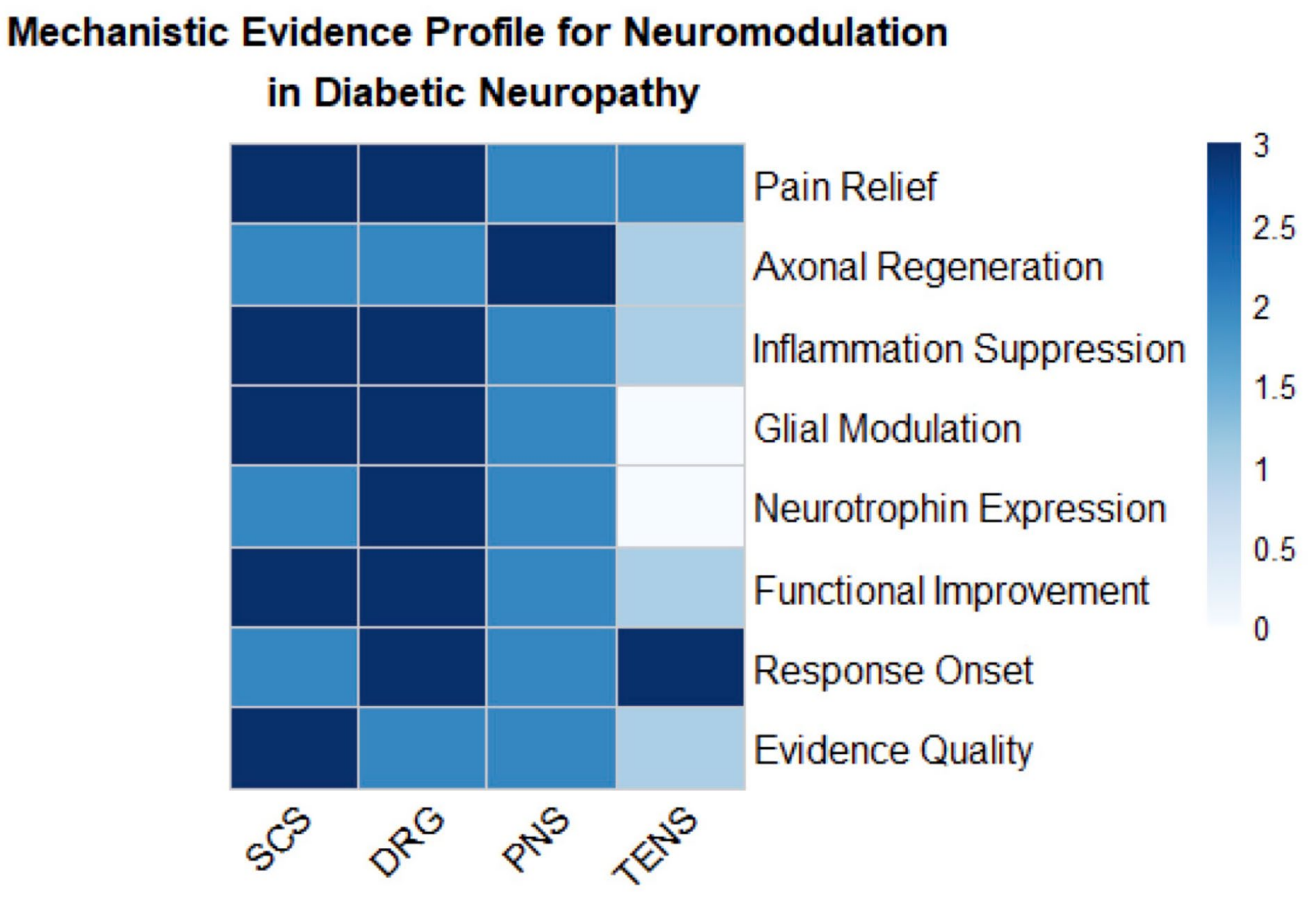
Comparative mechanistic engagement of neuromodulation therapies in diabetic neuropathy. This heatmap illustrates the extent to which SCS, DRG stimulation, PNS, and TENS engage eight key biological mechanisms implicated in DPN. The scoring system reflects the strength of available evidence rather than effect size alone: 3 (Strong): Mechanism supported by randomized controlled trials (RCTs) or robust clinical biomarker data. 2 (Moderate): Mechanism supported by uncontrolled clinical studies or consistent preclinical data across multiple models. 1 (Limited): Mechanism supported by isolated preclinical findings or theoretical extrapolation. 0 (None/Unknown): No current evidence supports this mechanism for this specific modality.

This layered understanding sets the stage for the mechanism-focused heatmap presented in Figure 3, which maps each neuromodulation therapy against eight biological mechanisms, from glial inhibition to neurotrophic factor upregulation, using evidence-weighted scoring. SCS and DRG consistently score high for multisystem modulation, while PNS shows particularly strong effects on structural repair. By contrast, TENS appears limited in scope, offering primarily short-term modulation of sensory pathways. The heatmap reinforces the concept of modality-specific mechanisms, a critical insight when aligning treatment choice with patient goals (e.g., rapid pain control vs. long-term regeneration).

To maximize the regenerative potential of PNS in DPN, careful consideration of stimulation parameters and device selection is required. Modern percutaneous systems (e.g., SPRINT PNS, Nalu, Curonix, StimRouter) offer sophisticated programming that can be tailored to target specific pathophysiological mechanisms. Current evidence suggests that lower-frequency stimulation (e.g., 2–10 Hz) may preferentially enhance local microvascular perfusion and upregulate neurotrophic factors, thereby facilitating Schwann cell activity and axonal repair. Conversely, high-frequency waveforms (e.g., 100 Hz and above) or varied pulse widths may be more effective for immediate blockade of nociceptive transmission. Future restorative trials must systematically compare low-frequency, high-pulse-width paradigms against high-frequency platforms to establish an optimal ‘regenerative dose’ that concurrently manages pain and drives structural nerve repair (48–50).

### 3.5 Physiological and regenerative mechanisms

Neuromodulation therapies exert their effects through a constellation of mechanisms that may in fact may help explain symptom relief. While the earliest and most foundational model, the gate control theory, attributes pain inhibition to the activation of large-diameter Aβ fibers that modulate nociceptive input at the dorsal horn, this represents only a part of a more expansive biological story (22). Increasingly, neuromodulation is recognized to impact diverse molecular and cellular pathways, including neuroinflammation, glial reactivity, and neurotrophic signaling cascades, which collectively support both functional improvement and structural repair (36).

Broader mechanistic effects, including Schwann cell activation, axonal regrowth, and myelin repair, are depicted in a cross-sectional schematic of peripheral nerves (Figure 2). Three fascicle regions, symbolically mapped to the tibial, sciatic, and sural nerves—highlight how various neuromodulation modalities target nerve architecture at different levels. For instance, DRG stimulation is associated with localized suppression of satellite glial cell activation and downregulation of pro-inflammatory cytokines, while SCS and PNS demonstrate enhancements in axonal sprouting, Schwann cell activity, and myelin integrity (51). These features are not merely pre-clinical or theoretical evidence, as some clinical studies have also corroborated improvements in IENFD, NCV, and histological markers of regeneration following successful neuromodulation (28). A few representative examples include a prospective open-label study where high-frequency SCS significantly increased IENFD after six months, along with improvements in thermal thresholds and gait stability (28). Additionally, pre-clinical models demonstrated that DRG stimulation reduced TNF-*α* and IL-1β expression in DRG and attenuated spinal glial activation, providing mechanistic support for the analgesic and neuroprotective effects (16). Collectively, these findings suggest that neuromodulation may offer not just symptomatic relief, but also disease-modifying potential through modulation of neuroinflammatory and regenerative pathways.

At the supraspinal level, SCS has been shown to increase nitric oxide synthase expression, thereby improving regional blood flow and reducing oxidative stress in dorsal horn neurons (16). Moreover, functional MRI and electrophysiological analyses revealed activation of pain modulation centers such as the thalamus and periaqueductal gray, indicating a role in both sensory gating and affective regulation of pain signals (4, 16).

By contrast, DRG stimulation exerted its effects at the junction of the peripheral and central nervous systems. DRG stimulation modulates afferent activity at specific dermatomes, resulting in precise sensory targeting with minimal off-target activation. Mechanistic studies support its association with reduced excitatory neurotransmitter release, downregulation of glial reactivity, and upregulation of genes involved in neuronal survival and synaptic stabilization (52, 53). PNS, although more peripheral in scope, plays a crucial role in neuroregeneration. In addition to dampening nociceptive input, it stimulates axon regeneration and remyelination through the recruitment of Schwann cells and local neurotrophic factor release (5). These findings were substantiated by increased NCV and histological recovery in affected nerve territories following therapy (54).

The modality-specific pathways highlighted in the peripheral nerve schematic underscore neuromodulation’s multi-layered therapeutic effects, from spinal reflex modulation to structural nerve repair (Figure 2). These insights position neuromodulatory therapies not merely as analgesic tools, but as potential disease-modifying interventions capable of reversing or slowing the structural and functional decline characteristic of DPN.

As with any neuromodulatory strategy, patient selection plays a pivotal role in therapeutic success. Evidence suggests that patients with preserved nerve conduction and shorter disease duration, typically within 5 years of symptom onset, experience the greatest benefits in both pain reduction and functional improvement (37). This underscores the importance of timely referral and early intervention, and underscores the degenerative nature of DPN that may represent critical windows for intervention, and potentially a point at which therapies can no longer be effective due to a threshold of nerve damage severity.

The ability to predict patient response is also evolving. Emerging biomarkers, such as IENFD and corneal nerve morphology, correlate with treatment efficacy, especially in SCS responders. In addition, functional MRI has shown promise in identifying somatosensory activity patterns that may stratify patients by neuromodulation responsiveness, laying the groundwork for more personalized therapy pathways. More work is needed in this space.

Innovative care models are beginning to be incorporated with multimodal approaches, for example, pairing DRG stimulation with physical therapy or anti-inflammatory regimens, to reduce central sensitization and minimize opioid dependence (55). These combinations reflect a shift toward synergistic, mechanism-based treatment planning, rather than a single-modality mindset.

The future of neuromodulation also lies in technological innovation. Machine learning–driven closed-loop SCS systems, capable of analyzing predictive pain models in real-time, are beginning to dynamically adapt stimulation parameters based on user behavior, sleep cycles, and physiological stress. These smart systems may substantially enhance therapeutic precision, especially in patients with fluctuating pain profiles or comorbid conditions.

Early intervention, aided by accurate biomarkers, improved functional diagnostics for small fiber neuropathy, and adaptive technologies, hold the potential to enable preserved nerve integrity, halt progression of neurodegeneration, and improve long-term outcomes, including quality of life (56). Both SCS and DRG stimulation have demonstrated durable efficacy and neuroprotective benefits, particularly when applied in the early stages of DPN (35).

These layered clinical and mechanistic distinctions can be observed through the temporal progression of therapeutic effects across neuromodulation modalities (30, 35). SCS and DRG produce robust pain relief within 3 months, followed by regenerative effects (Figure 3)—such as increased conduction velocity and IENFD emerging between 6 and 12 months. PNS, while also demonstrating moderate pain relief and regenerative gains by 12 months, exhibited greater variability based on lead placement and baseline nerve integrity. By contrast, TENS provided relatively rapid but short-lived analgesia, with minimal evidence for long-term structural repair. This reinforces the value of mechanism-informed modality selection, allowing clinicians to tailor therapy based on the urgency of symptom control versus the goal of long-term neural recovery.

As neuromodulation continues to evolve, so too do the systems that deliver it. Home-based data uploads, remote clinician programming, and user-guided feedback interfaces are becoming integral to care delivery, bridging the gap between advanced neuromodulation technologies and real-world patient accessibility.

## 4 Discussion

DPN remains among the most prevalent and disabling complications of diabetes mellitus, contributing significantly to patient morbidity through chronic pain, sensory loss, and increased risk of falls, foot ulcers, and depression. It is also a research area that remains critically under-funded, leading to a dearth of mechanistic research findings compared to other neurodegenerative conditions like Alzheimer’s, which impacts fewer people (57). Beyond the skin, numerous studies have revealed involvement of small fiber neuropathy in numerous organs and tissues, many with important metabolic functions, such as muscle, heart, pancreas, intestine, and adipose (58). Despite the availability of various pharmacological agents—including anticonvulsants, serotonin-norepinephrine reuptake inhibitors, analgesics, and topical agents—clinical efficacy to treat or reverse any DPN remains suboptimal. Fewer than half of patients achieve meaningful pain relief with painful DPN, and treatment discontinuation due to adverse effects such as sedation, dizziness, and gastrointestinal symptoms is common. Considering these limitations, neuromodulation has gained traction as a compelling alternative that may offer both symptomatic and structural benefits.

### 4.1 Translating efficacy to clinical practice

Rather than merely mirroring the analgesic metrics established in clinical trials, the real-world value of implantable neuromodulation lies in its broader impact on patient care and quality of life (23, 27, 59). Real-world evidence indicates that SCS and DRG stimulation facilitate decreased opioid reliance and significant improvements in mood, sleep, and overall independence (40). Most crucially, because these modalities show potential for structural nerve repair, demonstrated by increased IENFD and enhanced corneal nerve branching, neuromodulation is actively being reframed from a last-resort palliative option into a disease-modifying therapy suitable for early-stage or pharmacoresistant patients (60, 61). Additional studies with larger cohorts and deeper molecular phenotyping will inform future clinical applications of neuromodulation for DPN and potentially other neuropathies.

### 4.2 Real-world evidence and observational outcomes

While randomized controlled trials establish foundational efficacy, real-world evidence and post-marketing registries are crucial for validating the long-term utility of neuromodulation in routine clinical practice. Observational cohorts and broad registry data consistently mirror the robust outcomes observed in highly controlled environments. In real-world DPN cohorts, patients report significant, sustained reductions in visual analog scale (VAS) pain scores alongside profound decreases in oral analgesic and systemic opioid consumption. Furthermore, post-marketing data frequently highlights meaningful improvements in secondary outcomes, including sleep architecture, functional mobility, and overall health-related quality of life. These observational findings confirm that the analgesic, and potentially disease-modifying, benefits of modalities like SCS and DRG stimulation are durable outside of the trial setting, reinforcing their reliability as long-term therapeutic interventions (25, 62).

Beyond randomized controlled trials, an expanding body of real-world evidence supports the effectiveness of neuromodulation for painful diabetic neuropathy. Long-term observational studies of spinal cord stimulation have demonstrated sustained pain relief extending up to 8–10 years in selected patients, suggesting durable clinical benefit outside controlled research settings (24). Similarly, multicenter retrospective studies of dorsal root ganglion stimulation have reported meaningful reductions in pain and improvements in function among patients with refractory peripheral neuropathic pain, supporting the generalizability of neuromodulation across diverse clinical populations (25, 36).

Real-world analyses have also demonstrated broader healthcare benefits. In a recent retrospective cohort study, spinal cord stimulation for painful diabetic neuropathy was associated with favorable long-term outcomes and reduced healthcare burden (19). Furthermore, post-marketing economic analyses from the SENZA-PDN program found that patients receiving 10-kHz spinal cord stimulation incurred substantially lower annual healthcare costs than those receiving conventional medical management, primarily driven by reductions in healthcare utilization and hospitalizations (63).

Collectively, these observational and post-marketing data complement randomized trial findings by demonstrating that the benefits of neuromodulation can be reproduced in routine clinical practice. However, additional prospective registries and long-term real-world studies are needed to better define patient selection criteria, device performance, durability, and potential regenerative outcomes across broader patient populations.

### 4.3 Emerging neuromodulation modalities for DPN

In addition to the more established modalities, several emerging neuromodulation strategies are being explored for the treatment of DPN, offering potentially complementary or alternative mechanisms of action.

Scrambler therapy is a non-invasive technique that introduces synthetic “non-pain” signals via surface electrodes. It has shown short-term promise in reducing pain, though sustained efficacy beyond 3 months remains uncertain due to limited longitudinal data (64). Similarly, vagus nerve stimulation (VNS) and ultrasound therapy are under active investigation. Preclinical studies on VNS suggest it may modulate systemic inflammation through activation of the cholinergic anti-inflammatory pathway, while simultaneously enhancing axonal integrity and neuroimmune balance (65). Although these approaches remain investigational, they expand the therapeutic landscape and may serve specific patient populations not suited for implantable devices.

Therapeutic ultrasound is emerging as a potential non-invasive option for DPN, hypothesized to promote nerve repair through both thermal and non-thermal mechanical effects. Preclinical studies suggest that low-intensity therapeutic ultrasound (LITUS) may accelerate axonal regeneration and remyelination by upregulating brain-derived neurotrophic factor (BDNF) and enhancing local microvascular perfusion (66). In small clinical pilot studies, therapeutic ultrasound has demonstrated short-term improvements in pain scores and sensory NCV compared to sham treatment (67). However, unlike the robust, durable datasets supporting SCS, evidence for ultrasound remains limited to small, single-center trials with variable stimulation parameters, necessitating larger randomized studies to establish clinical viability.

### 4.4 Safety profile and considerations

Adverse events varied significantly by treatment modality, with non-invasive therapies (e.g., TENS, FREMS) demonstrating a superior safety profile characterized by zero reported infections and only minor skin irritation. For invasive neuromodulation (SCS and DRG), the incidence of surgical site infections generally ranged from 1.0 to 5.0%, though one older, small-cohort study reported rates as high as 10% (68). Beyond infection, device-related complications were a primary driver for re-intervention; lead migration was a frequently cited hardware complication, contributing to explant rates that ranged from approximately 2.3% in 24-month 10 kHz SCS trials to as high as 28% in 5-year follow-ups of tonic SCS due to combined loss of efficacy and technical failure.

### 4.5 Limitations in current evidence

Despite encouraging results, much of the evidence remains limited by small sample sizes, lack of blinding, or industry sponsorship that may carry bias. Many trials use open-label or crossover designs, introducing performance and detection bias (69). Inconsistent patient selection further hampers reproducibility and generalizability.

The current evidence base for neuromodulation in DPN is constrained by distinct methodological limitations regarding funding sources and blinding protocols. A significant proportion of the high-impact data, particularly for 10 kHz SCS, is derived from industry-sponsored trials (e.g., SENZA-PDN), raising potential concerns regarding sponsorship bias compared to investigator-initiated studies. Furthermore, adequate blinding remains a pervasive challenge in SCS research. Traditional tonic SCS relies on patient-perceived paresthesias (tingling) to confirm coverage, rendering true double-blinding against a sham control impossible. While 10 kHz SCS is paresthesia-free, the major trials utilized an open-label design comparing the implant against Conventional Medical Management (CMM), meaning subjects were aware of their treatment assignment. In contrast, DRG stimulation allows for highly specific dermatomal targeting which may facilitate better-controlled comparisons, and non-invasive modalities (e.g., TENS) were successfully able to utilize sham-controlled designs; however, the invasive DRG literature currently relies heavily on retrospective or observational data rather than the large-scale RCTs seen in the SCS domain.

A major gap is the absence of head-to-head RCTs comparing SCS, DRG, and PNS. Clinicians must rely on single-modality studies or indirect comparisons, complicating treatment decisions and increasing mismatch risk. Most trials also focus narrowly on pain, rarely capturing regenerative, functional, or quality of life/economic outcomes (70).

Robust, longer-term trials with standardized inclusion criteria and validated mechanistic biomarkers are needed. These should prioritize outcomes that reflect real-world goals, such as quality of life, functional recovery, and long-term nerve health.

### 4.6 Mechanisms of action and regenerative potential: still poorly understood

Beyond traditional gate control, neuromodulation affects neuroimmune signaling, vascular dynamics, and CNS plasticity—suggesting a broader therapeutic scope (36). However, key mechanisms remain poorly defined in humans. Clinical trials rarely assess glial activity, cytokine suppression, or neurotransmitter shifts. Biomarkers like TNF-*α*, IL-6, and NGF show promise but are inconsistently measured (51).

Advanced tools, like fMRI, PET, and corneal confocal microscopy, could track structural changes but remain underutilized in neuromodulation trials (71). The absence of these modalities leaves a critical gap between preclinical promise and clinical application. Mechanistic engagement (Figure 3) shows broad central and peripheral effects for SCS and DRG, while PNS shows strong axonal repair potential. TENS offers limited mechanistic depth.

Preclinical data support these patterns: both DRG and SCS modulate glial and neuronal gene expression. DRG stimulation influences inflammatory cytokine signaling at the dorsal root level, while SCS, particularly differential target multiplexed SCS, has been shown to regulate neuroimmune interactions and alter transcriptional activity in both neurons and glia (16, 36, 72). These signals suggest regenerative potential, but large, biomarker-integrated trials are needed to validate pathways and link them to clinical outcomes.

### 4.7 Bridging the gap: toward mechanistically-informed clinical trials

To translate mechanistic insights into practice, future trials must incorporate molecular, imaging, and electrophysiological biomarkers to objectively measure regeneration. Stratifying participants by nerve integrity (large vs. small fiber involvement, motor versus sensory symptoms, for example), diabetes duration and management, and other potentially confounding comorbidities (ex: cardiovascular disease) can refine selection and improve efficacy signals.

Studies should also prioritize early-stage DPN, when nerve injury may still be reversible. Multidimensional outcomes, including autonomic function, glycemic variability, and microvascular health, could complement standard pain metrics.

Ultimately, regenerative endpoints such as IENFD, nerve conduction, myelin thickness, and sensory recovery will be key to determining whether neuromodulation modifies disease or merely relieves symptoms like pain.

### 4.8 Future directions and clinical integration

With growing evidence, neuromodulation could become a core treatment for DPN. Integration into care requires longitudinal data and collaboration across endocrinology, neurology, and pain medicine. Early candidate identification, especially in stages where reversibility may prove to be a possibility, is critical.

With this said, an important unresolved clinical question is the optimal timing of neuromodulation in DPN. Current guidelines reserve implantable neuromodulation for patients with painful neuropathy refractory to conventional medical management (41). However, accumulating evidence suggests that patients with shorter disease duration and more preserved nerve function achieve greater improvements in pain and functional outcomes following neuromodulation (35). These observations raise the possibility that a critical therapeutic window may exist before substantial axonal degeneration and loss of regenerative capacity occur. If it is ultimately shown that neuromodulation truly exerts neuroprotective and regenerative effects through modulation of neuroinflammation, glial activation, and neurotrophic signaling, earlier intervention may maximize its ability to preserve nerve integrity and alter disease progression (15, 26, 34). At present, evidence remains insufficient to support routine implantation at the time of diagnosis or in asymptomatic individuals because the available clinical trials have primarily enrolled patients with painful, refractory DPN (21, 23, 24, 73). Future prospective trials should directly investigate whether earlier neuromodulation can improve long-term neurological outcomes and modify the natural history of DPN.

AI and wearable devices could help tailor therapy in real time, adjusting stimulation based on patient-reported outcomes, biometrics, or activity patterns (74). These tools may improve adherence, detect nonresponse early, and decentralize trials to include patients with limited mobility.

To support clinical adoption, trials must capture real-world outcomes—PROMs, cost-effectiveness, and quality of life—not just pain reduction. Mechanism-informed therapy, personalized via digital tools, may ultimately expand access and improve long-term results.

Finally, the regenerative mechanisms highlighted in this review may hold relevance beyond diabetes alone. Small fiber DPN also occurs with aging, chemotherapy, long COVID, and even metabolic disease outside of diabetes (obesity, pre-diabetes), the latter increasingly recognized as contributing to an epidemic of neuropathy (73, 75). As such, validating neuromodulation as a disease-modifying therapy in DPN could establish a framework for treating this broader ‘epidemic of neuropathy,’ extending the therapeutic potential of these modalities to millions of non-diabetic patients.

### 4.9 Clinical practice guidelines and health economic considerations

Given the growing body of high-quality evidence, clinical practice paradigms are actively shifting. A major milestone occurred in 2021 when the U. S. Food and Drug Administration (FDA) formally approved high-frequency (10 kHz) SCS for the treatment of refractory painful DPN. Consequently, consensus guidelines from specialized interventional pain societies have updated their algorithms to strongly recommend SCS as a treatment option for patients who have failed to achieve sufficient relief with standard pharmacologic classes, such as gabapentinoids or tricyclic antidepressants. Notably, these guidelines highlight high-frequency (10 kHz) stimulation as a preferred modality, citing its ability to provide paresthesia-free relief and superior outcomes in neurological function compared to traditional low-frequency approaches (41).

Crucially, this evolving mechanistic understanding necessitates a fundamental reevaluation of intervention timing. Rather than reserving neuromodulation as a late-stage ‘salvage’ therapy for patients who have suffered with refractory DPN for decades, the current evidence strongly supports earlier utilization. Deploying devices such as SCS, DRG, or PNS shortly after the failure of first-line pharmacotherapy, and prior to the onset of irreversible axonal degradation and central sensitization, may effectively capitalize on a critical regenerative window. By intervening earlier in the disease course, clinicians have the profound opportunity to not only provide superior analgesia but to actively halt disease progression and preserve neural architecture (63, 76).

In addition to clinical endorsement, addressing economic barriers is critical for broader real-world adoption. While the upfront cost of neuromodulation implantation is high, recent economic analyses suggest long-term savings. Data from the SENZA-PDN randomized controlled trial demonstrated that patients treated with 10 kHz SCS incurred significantly lower annual healthcare costs compared to those on conventional medical management (mean annual cost: $6,300 vs. $9,532, respectively) (77). This approximate 34% reduction in cost for the SCS group was driven primarily by decreased hospitalizations and healthcare resource utilization, suggesting that effective neuromodulation may be cost-protective over time despite the initial investment (77).

## 5 Conclusion

Neuromodulation represents a significant advancement in the management of DPN, though the evidence supports a clear distinction between long-term efficacy and neuroregenerative properties across modalities. While non-invasive approaches like TENS offer accessible but transient symptomatic relief, implantable therapies, specifically SCS and DRG stimulation, have demonstrated robust, durable analgesia in refractory populations and are being investigated for sustained restoration of tissue innervation.

Crucially, emerging mechanistic data suggests that these invasive modalities extend beyond simple pain masking. By modulating glial activation, reducing neuroinflammation, and potentially upregulating neurotrophic factors, SCS and DRG stimulation appear to engage the fundamental biology of nerve repair. This “disease-modifying” potential marks a critical divergence from palliative pharmacotherapy.

However, realizing this potential requires a shift in research methodology. Future clinical trials must move beyond subjective pain scores to incorporate objective regenerative biomarkers—such as IENFD, corneal confocal microscopy, and functional neuroimaging. Only by validating these structural outcomes can we confirm whether neuromodulation truly reverses the trajectory of diabetic neuropathy or merely silences its symptoms. As the field evolves, rigorous, mechanism-based patient selection will be essential to transforming these technologies from last-resort options into targeted, restorative treatments.
